# Dietary Patterns and Obesity among Chinese Adults: Results from a Household-Based Cross-Sectional Study

**DOI:** 10.3390/ijerph14050487

**Published:** 2017-05-05

**Authors:** Yan Zou, Ronghua Zhang, Shichang Xia, Lichun Huang, Jia Meng, Yueqiang Fang, Gangqiang Ding

**Affiliations:** Zhejiang Provincial Center for Disease Control and Prevention, 3399 Binsheng Road, Hangzhou 310051, China; zouyan0573@163.com (Y.Z.); rhzhang@cdc.zj.cn (R.Z.); lchhuang@cdc.zj.cn (L.H.); jmeng@cdc.zj.cn (J.M.); yqfang@cdc.zj.cn (Y.F.); yingyang900@yeah.net (G.D.)

**Keywords:** dietary pattern, obesity, beverage

## Abstract

The key dietary pattern other than dietary factors influencing obesity has been reported by several large epidemiological studies. This study was carried out between 2010 and 2012 including 1613 adult residents in Zhejiang Province. Dietary patterns were extracted by factor analysis based on 24-h dietary recall. Associations with dietary patterns and obesity were examined and adjusted for age and gender by logistic regression. Five dietary patterns were identified by factor analysis with their eigenvalues greater than 1: ‘cereal, animal, and plant food’, ‘high protein food’, ‘plant food’, ‘poultry’, and ‘beverage’. After adjustment for age and gender, the ‘cereal, animal, and plant food’ and ‘beverage’ pattern was associated with obesity (OR = 2.924, 3.257; 95% CI = 1.147–7.463, 1.372–7.692). In conclusion, ‘cereal, animal, and plant food’ and ‘beverage’ dietary patterns may be associated with increased risk of obesity. ‘Cereal, animal, and plant food’ dietary patterns may be associated with increased risk of obesity resulting from increased total energy intake by increased protein and fat intake; while a ‘beverage’ dietary pattern may be associated with increased risk of obesity resulting from increased total energy intake by increased carbohydrate intake. The findings are valuable in targeting future nutrition education.

## 1. Introduction

During the global nutrition shift period, obesity began to dominate the globe. Urban and rural areas from sub-Saharan Africa and South Asia’s poorest countries to the higher income countries have been shown to have experienced rapid increases in overweight and obesity prevalence [1]. In China, obesity rates across all age and gender groups have risen greatly in the past two decades [2]. The implications of these trends of obesity for health, quality of life, productivity, and health care costs are crucial.

A dietary pattern was defined as the number of various types of food in the diet and the proportion of the diet [3]. The dietary pattern approach, which takes into consideration possible interactions between nutrients or food items, could capture some of the complexity of diet that is frequently lost in the single-nutrient-based analyses [4,5] and may provide insightful information on its relationship with obesity risk factors. Dietary patterns were population specific and were influenced by sociocultural factors and food availability [6]. The relationship between dietary factors and chronic disease among adults living in China has been reported [7,8], and a study conducted in China reported that dietary patterns may affect the likelihood of metabolic syndrome [9].

This paper documents major dietary patterns under the current socio-economic environment, and describes the association between each dietary pattern and the risk of obesity, with a focus on the proportion of three macronutrients of each dietary pattern to explore the risk of being obese. It provides a comprehensive examination of the state of the current knowledge on the dietary dimension of Zhejiang Province, which is located in the southern part of Yangtze River Delta of the southeast coast of China, and may help promoting the health authorities in targeting campaigns about improving dietary habits to groups with unhealthy dietary habits.

## 2. Materials and Methods

### 2.1. Subjects and Principles

Adults from cities, townships, and residential villages in Zhejiang Province were the subjects of this study. A stratified cluster sampling technique was employed in the present cross-sectional study. Based on socioeconomic characteristics, two cities, two townships, and two residential villages were selected via stratified random sampling and where the investigation was conducted. In every sampling unit, 450 households were selected by random sampling method according to the household registration information. Then, every member of the sampled household was interviewed, with 5577 adults in total (2535 male and 3042 female subjects).

The participants were divided into two groups as a dietary survey group and non-dietary survey group. The first 30 households were selected among every sampling unit as the dietary survey group, and the other households were the non-dietary survey group. Dietary data collected by three consecutive days of 24-h dietary recall investigation were used to analyze the dietary patterns in this study.

All procedures performed in studies involving human participants were in accordance with the ethical standards of the institutional and/or national research committee and with the 1964 Helsinki declaration and its later amendments or comparable ethical standards. Informed consent was obtained from all individual participants included in the study. Research protocols were approved by the Zhejiang Provincial Center for Disease Control and Prevention (Ethic approval code: T-043-R-2010).

### 2.2. Dietary Information Collection

During home visits spanning three days, dietary data were collected through interviews with each household member of the sampling household, including rice and its products, wheat flour and its products, tubers, bean products, dark colored vegetables, light colored vegetables, pickled vegetables, pork, poultry, milk and dairy products, eggs, fish and shrimp, vegetable oil, and beverages. Energy and nutrient intake was calculated using three consecutive days of 24-h dietary recall in conjunction with the China Food Composition Table published in 2002 [10]. The questionnaire was administrated face to face by trained staffs through door to door interview. We have checked the data before analysis and excluded any implausible results, e.g., energy intake <500 kcal or >5000 kcal per day.

### 2.3. Anthropometric Measurements

Height was measured without shoes to the nearest 0.2 cm using a portable SECA stadiometer, and weight was measured without shoes and in light clothing to the nearest 0.1 kg on a calibrated beam scale. BMI (Body Mass Index) was calculated by weight (kg)/height (m^2^). According to “Guidelines for Prevention and Control of Overweight and Obesity in Chinese Residents”, we chose 28.0 kg/m^2^ as the cut-off for obesity. BMI was classified using underweight, normal weight, overweight, and obese categories [11].

### 2.4. Extraction of Dietary Patterns

Dietary patterns were identified using principal component analysis (the FACTOR procedure) in SAS with varimax rotation (SAS Institute Inc. SAS/STAT 9.2, Cary, NC, USA). To test the analysis, we used Bartlett test of sphericity (*p* < 0.001) and the Kaiser–Mayer–Olkin tests (0.633), denoting statistically correlated variables and adequate sampling size. Principal component analysis is a data driven technique that reduces the dimension of the data and groups correlated variables, to form new components. The number of patterns identified was determined by use of the scree plot and the interpretability of each of the patterns. The coefficients defining the components are called factor loadings and describe the correlation between each food group and the components (Table 1 and Table 2). Based on the reported method in the literatures [12,13], food groups with factor loadings of 0.30 or higher were considered as being strongly related to the particular pattern, and were applied in the characterization of the patterns. For a dietary pattern in which the food groups with factor loadings of 0.30 were less than three kinds, the first three food groups according to factor loadings were applied in the characterization of that pattern. The mean factor score for each pattern is zero. Positive factor scores indicate higher consumption of foods and in that pattern and negative factor scores indicate low consumption.

### 2.5. Statistical Methods

We calculated factor scores for five patterns and used them as the target variables. The score of each dietary pattern for each individual was obtained by multiplying the intake of each food with the factor scores. As continuous variables were not normally distributed—such as protein, fat, and carbohydrates—they were described as 25th (Q1) and 75th (Q4) percentiles. The differences of protein, fat, and carbohydrates between Q1 and Q4 were evaluated by nonparametric test (Mann–Whitney test). We used logistic regression model to examine the association between dietary patterns and obesity, and adjusted for gender and age. We also used nominal regression model to examine the association between dietary patterns and BMI categories, and adjusted for gender and age. Data processing and statistical analyses were performed using SAS9.2 software (SAS Institute). All tests were two-sided and the level of significance set at *p* < 0.05.

## 3. Results

### 3.1. Demographic Characteristics and Dietary Patterns

A total of 1613 adults were participated in this dietary pattern study (755 male and 858 female). The average age was 53.6 ± 14.8 years. The prevalence rate of ‘underweight’, ‘normal weight’, ‘overweight’, and ‘obesity’ was 6.6, 53.3, 31.0, and 9.1%, respectively. The distribution of participants’ characteristics by dietary patterns is shown in Table 3. There were no significant differences among the five dietary patterns on age, education, smoking, and income (*p* > 0.05).

**Figure 1:**
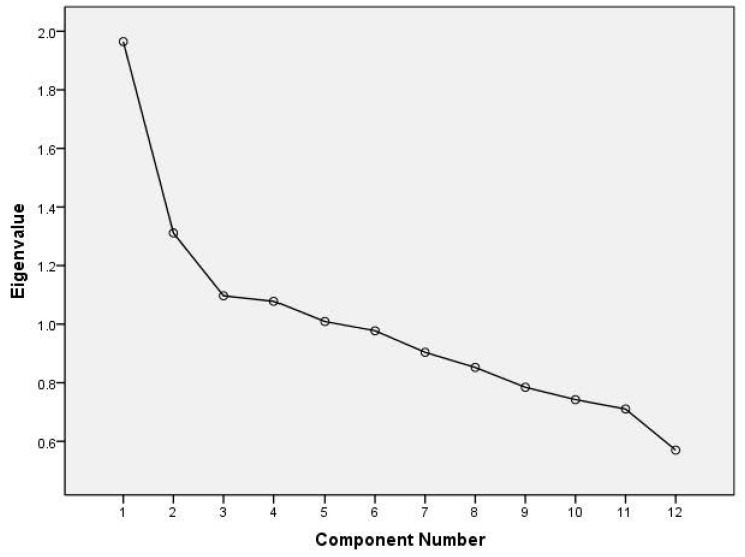
Scree plot for identification of dietary patterns (components) by principal component analysis. Food intakes (g/day) were aggregated into 12 food groups and used as input variables. Factors considered appropriate for patterns shown in Table 1 are the five factors with eigenvalues >1.

We extracted five distinct dietary patterns with eigenvalues above 1 from the scree plot, as well as factor loadings (Table 1 and Figure 1); these five patterns accounted for 53.8% of the total variation in food intakes. Five patterns were identified and named accordingly: a ‘cereal, animal, and plant food’ pattern with positive correlations with pork, vegetables, cereals, fish, and shellfish. A ‘high protein’ pattern had positive factor loadings for milk and dairy products, eggs, fish, and shellfish and fruits. A ‘plant food’ pattern positively correlated with beans, fruits, and nuts. Another two patterns were ‘poultry’ and ‘beverage’ patterns. The percentages of variation explained were 16.4, 10.9, 9.1, 8.9, and 8.4% respectively.

### 3.2. Risk of Being Obese Related to Each Dietary Pattern

Crude and adjusted odds ratio (OR) and confidence interval (CI) of all five dietary patterns for obesity are shown in Table 4, and the ‘beverage’ pattern was associated with significantly increased risk of obesity (OR = 1.285; 95% CI = 1.094–1.510). After adjustment for age, gender, education, smoking, total energy intake, household income, the ‘beverage’ pattern was associated with obesity (OR = 1.286; 95% CI = 1.095–1.511).

Crude and adjusted odds ratio (OR) and confidence interval (CI) of all five dietary patterns for BMI categories (underweight, normal weight, overweight, and obese) are shown in Table 5. ‘Cereal, animal, and plant food’ and ‘beverage’ patterns were associated with significantly increased risk of obesity (Odds ratio (OR) = 2.967, 3.077; 95% confidence interval (CI) = 1.238–7.143, 1.304–7.246). After adjustment for age, gender, total energy intake, the ‘cereal, animal, and plant food’ pattern and ‘beverage’ pattern were associated with obesity (OR = 2.924, 3.257; 95% CI = 1.147–7.463, 1.372–7.692).

### 3.3. Contribution Rate of Three Macronutrients of Each Dietary Pattern

The contribution rates of three macronutrients of each dietary pattern are shown in Table 6. The upper quartile (Q4) group of ‘cereal, animal, and plant food’, ‘plant food’, and ‘poultry’ pattern had higher energy intakes 1.78-, 1.45-, and 1.09-fold greater than the lower quartile (Q1) group (*p* < 0.01).

There were significant differences on the total energy between the lower quartile (Q1) and the upper quartile (Q4) of ‘cereal, animal, and plant food’, ‘plant food’, ‘poultry’, and ‘beverage’ pattern. Compared with the lower quartile (Q1) of ‘cereal, animal, and plant food’, ‘plant food’, and ‘poultry’ pattern, the upper quartile (Q4) of the three patterns showed significantly high intake of protein (*p* < 0.01), while the upper quartile of ‘beverage’ patterns showed low intake of carbohydrate(*p* < 0.01). Compared with the lower quartile (Q1) of ‘cereal, animal, and plant food’, and ‘poultry food’ pattern, the upper quartile (Q4) of the two patterns showed significantly high intake of fat (*p* < 0.01), while the upper quartile of ‘plant food’ and ‘beverage’ patterns showed low intake of fat (*p* < 0.01). Compared with the lower quartile (Q1) of the ‘cereal, animal, and plant food’ and ‘poultry’ patterns, the upper quartile (Q4) of the two patterns showed significantly low intake of carbohydrates (*p* < 0.01), while the upper quartile of ‘plant food’ and ‘beverage’ patterns showed high intake of carbohydrates (*p* < 0.01).

Compared with other dietary patterns, the Q4 group of ‘beverage’ pattern had higher proportion of carbohydrates and comparatively lower proportion of fat and protein that contributed to total energy than the Q1 group (53.2% ± 11.7 vs. 44.7% ± 11.4, *p* < 0.01). The Q4 group of ‘beverage’ pattern had higher energy intake of 1.19-fold than the Q1 group. The distribution of fat and dietary fiber intake by dietary patterns is shown in Table 7. Beverage dietary pattern had low fat intake from animal food (*p* < 0.05).

## 4. Discussion

The dietary pattern approach summarized nutrient and food intake to depict the whole diet and reflects dietary preferences and actual consumption, and thus identifies groups at nutritional risk. Using this method, we identified five distinct dietary patterns; namely, ‘cereal, animal, and plant food’ (rice, vegetables, pork), ‘high protein food’ (milk, eggs, fish), ‘plant food’ (beans, fruits, nuts), ‘poultry’ (chicken, duck), and ‘beverage’ (carbonated beverage, fruit drinks). Of these, ‘cereal, animal, and plant food’ and ‘beverage’ patterns were associated with a significantly increased risk of obesity. ‘cereal, animal, and plant food’ dietary pattern may be associated with increased risk of obesity resulting from increased total energy intake by increased protein and fat intake; while ‘beverage’ dietary pattern may associated with increased risk of obesity resulting from increased total energy intake by increased carbohydrate intake. These findings are important, as prevention of dietary style is of major importance in obesity.

Understanding the associations between the ‘cereal, animal, and plant food’ and ‘beverage’ dietary patterns and obesity may help to promote healthy changes in dietary behavior that might be neglected during daily life, with the aim of ensuring obesity prevention. Our results have important public health and nutritional implications, particularly given the emerging ‘beverage’ dietary pattern and its risks on obesity. ‘Beverage’ dietary pattern includes soft drinks, fruit juice, artificially sweetened beverages, and coffee. We have the common perception that the ‘beverage’ pattern can have a direct effect in the risk of obesity, because the ‘beverage’ pattern has a higher proportion of carbohydrates (monosaccharides and disaccharides) that contribute to total energy. This study helped us to understand it is one of the current five major dietary patterns that with poor diet quality, because different from the ‘cereal, animal, and plant food’, pattern. Beverage pattern could increase the risk of obesity resulting from increased total energy intake by increased carbohydrate intake. This study also suggested that ‘cereal, animal, and plant food’ dietary pattern may have imbalanced proportions of cereal, animal, and plant food and have higher fat and protein intakes than traditional Chinese dietary pattern, increasing the risk of obesity.

In Popkin’s study, the overall dietary quality was lower in low-calorie-sweetened, calorie-sweetened beverage consumers [14], and another study conducted in Saudi Arabia indicated that a higher intake of beverage is associated with poor dietary choices [15]. Dietary patterns also have been associated with health related and other lifestyle factors in previous studies [16,17]. This points toward the fact that less healthy dietary patterns are associated with unfavorable behavioral factors, which may be valuable knowledge in future strategies in promoting public health nutrition. Thus, future studies identifying demographic and lifestyle factors associated with certain dietary patterns are warranted to support the prevention strategies.

Most previous studies have focused on specific foods or nutrients in relation to obesity, but distinguishing the foods in a mixed diet that are responsible for the effect can be difficult. Factor analysis is a robust and meaningful technique for dietary pattern analysis and is useful for understanding the role of dietary patterns in health and disease [18]. This study found that the ‘cereal, animal, and plant food’ dietary pattern may increase the risk of obesity. Previous studies conducted in a Chinese National survey from 2002 have identified several dietary patterns like the ‘yellow earth’ or ‘traditional northern’ pattern—high in wheat, wheat products, and tubers; the ‘traditional southern’ pattern—high in rice, vegetables, seafood, pork, and poultry; and the ‘western’ pattern—high in beef, milk, juices, and nuts [19,20]. Recently, a study focused on childhood obesity reported that the modern dietary pattern and the traditional northern dietary pattern were associated with higher risk of obesity [21]. Another study focused on young Chinese women reported that the traditional ‘northern pattern’ was positively associated with general and abdominal obesity [22]. A longitudinal analysis of dietary patterns in Chinese adults from 1991 to 2009 indicated that increasing popularity of the modern high-wheat dietary pattern, a pattern associated with several energy-dense foods, is a cause of concern under rapid economic changes period in China [23]. However, subjective decisions such as the grouping of foods, the rotation methods, the number of components to be retained, and their subsequent labeling was different. Although the dietary patterns in the current study could not be compared directly with those of other studies because of the difference in the process (the grouping of foods, the rotation methods, the label of dietary pattern), the results of those reports were similar on the dietary pattern identification, but the ‘beverage’ pattern is newly identified in our study under the modern lifestyles in China. Carbohydrates provide a major source of energy in the diet, and the ‘beverage’ dietary pattern had low fat intake from animal food, and hence the type and amount of carbohydrates consumed is an important consideration for body weight control [24].

A study conducted in Spain found that a caloric beverage pattern dominated by intake of ‘soft drinks’ is related to general and abdominal adiposity in male adolescents [25]. Similarly, a study conducted in Colombia indicated that snacking and soda intakes are associated with development of adiposity in school-age children [26]. Combined with the previous studies, our findings also suggest that the ‘beverage’ dietary pattern is associated with increased risk of obesity.

This study included a large sample size which enables us to carry out factor analysis on dietary pattern and covariate analysis to explore the odds ratios for obesity. Strengths of this study also include that the data were collected covering city, township, and rural area adults, and thus not restricted to certain areas of urbanization. In addition, we assessed the patterns using three consecutive days of 24-h dietary recall method, which provided detailed information regarding the types of foods and beverages consumed.

There are several limitations to this study. First, the results cannot demonstrate a causal or resultant relationship among dietary patterns because of the cross-sectional design. Adults with obesity may have changed their diet following their clinician’s suggestions. If they then ate a healthy diet, the dietary influence detected may be the result, but not the cause, of obesity. Future prospective cohort studies are warranted to verify our findings. Second, in a review study, the percent of the variance explained by the dietary patterns ranged from 15% to 93% among 58 studies [27]. In the present study, the five dietary patterns identified by factor analysis accounted for 53.8% of the total variation in food intakes. Greater detail in food-use information may be desirable in determination of dietary patterns for more precise estimates of disease risk. Third, although our analysis included dietary pattern and demographic covariate variables that can affect obesity, residual confounding variables may still exist. We are forced to pre-specify the number of factors and although we used eigenvalues, scree plots, and interpretability, that we should accept such a decision is subjective [28]. In addition, the current BMI value is only a reference value of obesity, and visceral fat may be a more accurate indicator of obesity.

Our study is observational, and conclusions about causality cannot be drawn, but the results could serve as basis for dietary intervention guidelines, and could be translated in to public health recommendations.

## 5. Conclusions

In conclusion, five distinct dietary patterns were identified, namely, ‘cereal, animal, and plant food’, ‘high protein food’, ‘plant food’, ‘poultry’, and ‘beverage’. These provide a comprehensive examination of the state of the current knowledge on the diet-related dimensions in China. The ‘cereal, animal, and plant food’ and ‘beverage’ dietary patterns may be associated with increased risk of obesity. ‘Cereal, animal, and plant food’ may be associated with increased risk of obesity resulting from increased total energy intake by increased protein and fat intake; while the ‘beverage’ pattern may be associated with increased risk of obesity resulting from increased total energy intake by increased carbohydrate intake, and the findings are valuable in targeting future nutrition education.

## Figures and Tables

**Table 1:** Factor loadings of food groups in the five dietary patterns identified (loading equal to and above 0.30 are in bold).

Food Groups	Dietary Pattern				
	Cereal, Animal, and Plant Food	High Protein Food	Plant Food	Poultry	Beverage
Cereals	**0.603**	−0.005	−0.012	0.181	0.190
Tubers	−0.080	0.169	0.277	−0.491	−0.222
Beans	0.195	−0.268	**0.735**	0.003	−0.115
Vegetables	**0.663**	0.140	0.210	−0.249	−0.057
Fruits	−0.201	**0.398**	**0.622**	−0.163	0.170
Nut	0.125	0.132	**0.389**	0.265	0.104
Pork	**0.739**	0.111	−0.007	0.075	−0.066
Poultry	−0.018	0.092	0.175	**0.813**	−0.131
Milk and dairy products	0.056	**0.697**	−0.027	0.093	−0.156
Eggs	0.047	**0.635**	0.031	−0.068	0.204
Fish and shellfish	**0.358**	**0.519**	0.094	−0.012	−0.123
Beverage	0.023	−0.011	0.051	0.013	**0.895**

**Table 2:** The most commonly consumed foods under each food group.

Dietary Pattern	Most Commonly Consumed Foods
Cereal, animal, and plant food	rice, vegetables, pork
High protein food	milk, eggs, fish
Plant food	beans, fruits, nuts
Poultry	chicken, duck
Beverage	carbonated beverage, fruit drink

**Table 3:** The distribution of participants’ characteristics by dietary patterns.

Participants’ Characteristics		Dietary Patterns					*p*
		Cereal, Animal, and Plant Food	High Protein Food	Plant food	Poultry	Beverage	
Age (years old)		51.9 ± 16.6	53.9 ± 13.7	54.3 ± 14.8	53.2 ± 15.3	50.8 ± 15.3	0.054
Gender (male:female)		1:1.20	1:1.37	1:1.10	1.02:1	1.17:1	0.065
Education (%)	Not to go to school	2.4%	3.9%	2.9%	4.2%	3.9%	0.13
	Illiteracy	11.0%	9.6%	8.1%	9.2%	3.3%	
	Primary school	27.9%	28.1%	30.2%	23.0%	28.9%	
	Junior middle school	29.0%	32.5%	33.1%	37.5%	41.4%	
	Senior middle school	16.1%	3.9%	4.2%	4.0%	4.6%	
	Junior college	8.0%	5.5%	5.0%	7.9%	2.6%	
	University or above	5.6%	3.9%	4.2%	4.0%	4.6%		
Occupation (%)	Housekeeping	15.8%	14.3%	13.1%	9.0%	9.2%	0.000
	Unemployed	4.3%	2.5%	2.9%	5.0%	3.3%	
	Retirees	25.2%	26.7%	33.9%	29.6%	22.4%	
	Professionals	5.9%	6.3%	6.0%	6.6%	5.9%	
	Clerks	5.4%	5.5%	3.9%	4.7%	5.3%	
	Service personal	11.3%	8.8%	10.5%	12.7%	8.6%	
	Agricultural production personnel	18.5%	22.9%	21.0%	17.4%	34.2%	
	Operator	4.0%	7.7%	2.1%	7.4%	7.9%	
	Other	9.7%	5.2%	6.6%	7.7%	3.3%	
Income (yuan, %)	5000	11.0%	11.8%	11.8%	11.3%	14.5%	0.384
	5000–9999	11.5%	12.9%	13.1%	7.4%	12.5%	
	10,000–14,999	16.4%	16.3%	15.7%	21.4%	17.1%	
	15,000–19,999	20.9%	15.4%	17.3%	16.9%	15.8%	
	20,000–24,999	21.4%	17.9%	16.3%	20.3%	19.7%	
	25,000–29,999	3.5%	6.3%	5.8%	4.5%	4.6%	
	30,000–34,999	5.9%	4.4%	5.2%	5.5%	3.3%	
	≥40,000	9.4%	14.9%	14.7%	12.7%	12.5%	
Smoking (yes, %)		23.8%	24.0%	24.9%	26.1%	30.7%	0.527

**Table 4:** Risk of being obese related to each dietary pattern.

Dietary Pattern	OR		95% OR		*p*
			Lower	Upper	
Cereal, animal, and plant food					
	Crude OR (95% CI)	1.125	0.961	1.317	0.141
	Adjusted OR (95% CI) *	1.125	0.932	1.358	0.221
High protein food					
	Crude OR (95% CI)	1.062	0.908	1.241	0.452
	Adjusted OR (95% CI) *	1.083	0.922	1.272	0.333
Plant food					
	Crude OR (95% CI)	0.980	0.837	1.147	0.799
	Adjusted OR (95% CI) *	0.958	0.810	1.132	0.611
Poultry					
	Crude OR (95% CI)	1.053	0.900	1.231	0.522
	Adjusted OR (95% CI) *	1.050	0.894	1.234	0.550
Beverage					
	Crude OR (95% CI)	1.285	1.094	1.510	0.002
	Adjusted OR (95% CI) *	1.286	1.095	1.511	0.002

* Adjusted for age, gender, education, smoking, total energy intake, household income.

**Table 5:** Risk of obesity according to BMI categories related to each dietary pattern by nominal regression.

Dietary Pattern	OR		95% OR		*p*
			Lower	Upper	
Cereal, animal and plant food					
	Crude OR (95% CI)	2.967	1.238	7.143	0.015
	Adjusted OR (95% CI) *	2.924	1.147	7.463	0.025
High protein food					
	Crude OR (95% CI)	1.538	0.672	3.521	0.308
	Adjusted OR (95% CI) *	1.515	0.658	3.484	0.326
Plant food					
	Crude OR (95% CI)	0.934	0.397	2.197	0.874
	Adjusted OR (95% CI) *	0.938	0.398	2.208	0.883
Poultry					
	Crude OR (95% CI)	0.722	0.308	1.695	0.455
	Adjusted OR (95% CI) *	0.756	0.315	1.815	0.531
Beverage					
	Crude OR (95% CI)	3.077	1.304	7.246	0.010
	Adjusted OR (95% CI) *	3.257	1.372	7.692	0.007

* Adjusted for gender and age.

**Table 6:** Contribution rate of three macronutrients in total energy between the upper (Q4) and lower (Q1) quartile of each dietary pattern, and the total energy rate of Q4/Q1.

Dietary Pattern	Protein	*p*	Fat	*p*	Carbohydrate	*p*	Total Energy	Total Energy Rate	*p*
	(%)		(%)		(%)		(kcal)	(Q4/Q1)	
Cereal, animal and plant food									
Q1	13.7 ± 4.2	<0.001	34.6 ± 11.4	<0.001	49.6 ± 11.9	<0.001	2162.2 ± 788.1	1.78	0.000
Q4	14.7 ± 4.0		35.3 ± 10.5		47.3 ± 11.2				
High protein food							2204.1 ± 944.7		
Q1	12.8 ± 3.5	<0.001	33.1 ± 11.5	0.001	52.6 ± 12.3	0.002		0.98	0.828
Q4	16.0 ± 4.0		36.1 ± 9.9		47.0 ± 11.1				
Plant food							2123.1 ± 849.9		
Q1	13.8 ± 4.3	<0.001	38.5 ± 12.3	<0.001	46.1 ± 13.4	<0.001		1.45	0.000
Q4	14.8 ± 3.5		33.6 ± 9.3		51.1 ± 10.3				
Poultry							2176.9 ± 900.2		
Q1	13.6 ± 3.9	<0.001	33.7 ± 10.6	<0.001	52.2 ± 11.6	0.247		1.09	0.001
Q4	15.3 ± 4.0		37.0 ± 10.3		45.6 ± 11.3				
Beverage							2189.14 ± 901.7		
Q1	16.2 ± 4.1	0.003	38.2 ± 10.4	0.298	44.7 ± 11.4	<0.001		1.19	0.000
Q4	12.7 ± 3.7		32.2 ± 10.2		53.2 ± 11.7				

**Table 7:** The distribution of fat and dietary fiber intake by dietary patterns.

Fat and Dietary Fiber Intake	Dietary Patterns					*p*
	Cereal, Animal, and Plant Food	High Protein Food	Plant Food	Poultry	Beverage	
Fat from animal food	36.94 ± 22.1	31.64 ± 16.6	33.64 ± 18.6	37.04 ± 17.9	31.44 ± 15.4	0.000
Fat from plant food	63.14 ± 21.1	68.84 ± 16.6	66.44 ± 18.6	63.04 ± 17.9	68.64 ± 15.4	0.000
Dietary fiber	12.14 ± 11.6	10.5 ± 5.7	11.9 ± 9.2	10.5 ± 6.1	11.4 ± 6.5	0.014
